# Discrimination of the Cognitive Function of Community Subjects Using the Arterial Pulse Spectrum and Machine-Learning Analysis

**DOI:** 10.3390/s22030806

**Published:** 2022-01-21

**Authors:** Hsin Hsiu, Shun-Ku Lin, Wan-Ling Weng, Chaw-Mew Hung, Che-Kai Chang, Chia-Chien Lee, Chao-Tsung Chen

**Affiliations:** 1 Graduate Institute of Biomedical Engineering, National Taiwan University of Science and Technology, Taipei 106, Taiwan; a0931355907@gmail.com (W.-L.W.); f68528@gmail.com (C.-K.C.); b0926184483@gmail.com (C.-C.L.); 2 Biomedical Engineering Research Center, National Defense Medical Center, Taipei 114, Taiwan; 3 Institute of Public Health, National Yang Ming Chiao Tung University, Taipei 112, Taiwan; gigilaskl@gmail.com; 4 Department of Chinese Medicine, Taipei City Hospital, Renai Branch, Taipei 106, Taiwan; DAI44@tpech.gov.tw; 5 General Education Center, University of Taipei, Taipei 100, Taiwan; 6 Department of Healthcare, Taipei Veterans Home, New Taipei City 110, Taiwan; ivlwh1128@gmail.com; 7 Institute of Traditional Medicine, National Yang Ming Chiao Tung University, Taipei 112, Taiwan

**Keywords:** dementia, pulse, spectral analysis, machine learning, community subjects, Mini-Mental State Examination

## Abstract

Early identification of cognitive impairment would allow affected patients to receive care at earlier stage. Changes in the arterial stiffness have been identified as a prominent pathological feature of dementia. This study aimed to verify if applying machine-learning analysis to spectral indices of the arterial pulse waveform can be used to discriminate different cognitive conditions of community subjects. 3-min Radial arterial blood pressure waveform (BPW) signals were measured noninvasively in 123 subjects. Eight machine-learning algorithms were used to evaluate the following 4 pulse indices for 10 harmonics (total 40 BPW spectral indices): amplitude proportion and its coefficient of variation; phase angle and its standard deviation. Significant differences were noted in the spectral pulse indices between Alzheimer’s-disease patients and control subjects. Using them as training data (AUC = 70.32% by threefold cross-validation), a significant correlation (*R*^2^ = 0.36) was found between the prediction probability of the test data (comprising community subjects at two sites) and the Mini-Mental-State-Examination score. This finding illustrates possible physiological connection between arterial pulse transmission and cognitive function. The present findings from pulse-wave and machine-learning analyses may be useful for discriminating cognitive condition, and hence in the development of a user-friendly, noninvasive, and rapid method for the early screening of dementia.

## 1. Introduction

Dementia encompasses neurodegenerative disorders that are characterized by the progressive loss of cognitive function and the ability to perform activities of daily living [1]. It gradually becomes a burdensome disease not only for affected individuals but also their families [2].

The standard diagnostic assessment of dementia includes history-taking, clinical examinations (e.g., neurological, mental state, and cognitive examinations), and an interview with a relative other than the informant. Recent guidelines also recommend computed tomography or magnetic resonance imaging of the brain to exclude structural causes for the clinical phenotype [1,2]. It has been reported that anywhere from 29% to 76% of patients with dementia or probable dementia are not diagnosed by primary-care clinicians [2]. Early identification of cognitive impairment through screening would allow patients and their families to receive care at an earlier stage in the disease process, potentially allowing discussions regarding health, financial, and legal decision-making while the patient is still legally capable [2].

Screening is designed to identify unrecognized or asymptomatic disease by administering tests that can be applied rapidly without the primary intention of being diagnostic [1,2,3]. Recent UK health policy has encouraged the opportunistic testing of older people attending primary care [1]. Screening of people with suspected dementia usually involves a brief test of cognitive function, informant questionnaires, or both, with a low score indicating a need for more in-depth assessments [1]. It has also been suggest that structural neuroimaging, genetic testing, and brief structured assessments (mainly using various questionnaires) can be used in dementia screening [2].

Alterations of the cerebral macrovasculature and microvasculature have been found in association with dementia [4]. These vascular changes can reduce cerebral perfusion and impair the ability to supply energy substrates and oxygen to active brain regions, and thus play a role in neuronal dysfunction and damage [5]. The induced atherosclerosis takes place not only in intracranial vessels but also in extracranial arteries such as the carotid, femoral, and coronary arteries [5,6].

Machine-learning techniques are already widely used to analyze various kinds of biological signals. The arterial pulse waveform transmits along the artery, and its characteristics are determined by the interaction between the pumping of blood by the heart and the arterial tree; it can therefore provide information about arterial wall stiffness [7,8,9]. Changes in the pulse waveform can be detected by noninvasive measurements, and various analysis methods (e.g., pulse-wave-velocity analysis [7] and frequency-domain analysis [10,11]) have been applied to the pulse waveform to evaluate changes induced by aging and various diseases [12,13,14,15,16,17,18,19]. Changes in the pulse waveform are often complex, and machine-learning analysis has the advantage of being able to capture subtle changes induced by physiological and pathological factors [20]. For example, arterial pulse-wave measurements, frequency-domain pulse analysis, and machine-learning analysis were used to distinguish vascular aging [10]. Another study applying similar methods demonstrated that using multilayer-perceptron analysis with frequency-domain pulse indices as features is highly effective at distinguishing between Alzheimer’s-disease (AD) patients and control subjects, with an accuracy of >80% and a particularly high specificity of >90% [11].

Based on our previous findings [11], the present study included community-dwelling subjects from two community sites. The Mini Mental State Examination (MMSE) score was used to define the cognitive condition of the subjects, and the aim was to verify if applying machine-learning analysis to spectral indices of the pulse waveform can discriminate between different cognitive conditions. In the machine-learning analysis, threefold cross-validation was performed to evaluate the training of the models. We also attempted to identify a relationship between the MMSE score and the prediction probability from the testing results of the machine-learning model. The present findings on the induced changes in the vascular properties and the pulse waveform indices may be useful for developing a method to aid the early screening of dementia.

## 2. Materials and Methods

**Figure 1:**
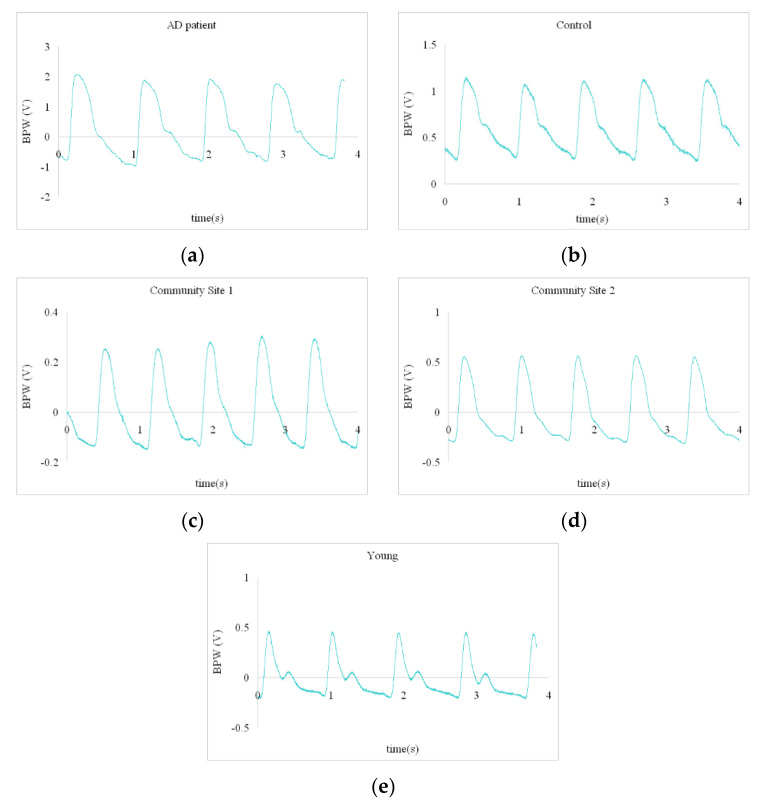
Typical measured pulse waveforms. (**a**) AD patient; (**b**) Control; (**c**) Community Site 1; (**d**) Community Site 2; (**e**) Young.

**Figure 2:**
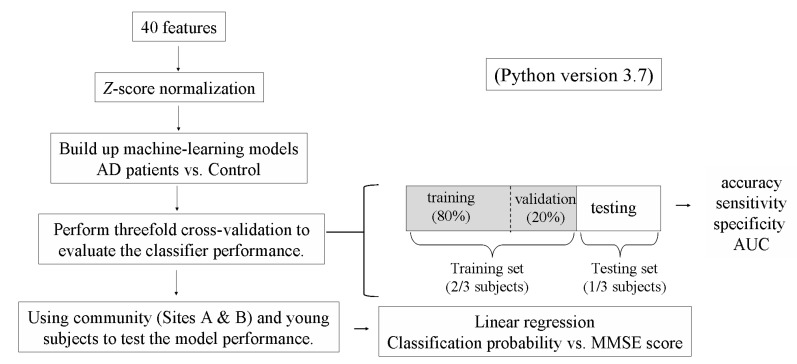
Procedure for information processing.

Details of the present experimental setup and the signal processing methods are available elsewhere [10,11,15]. BPW signal was noninvasively measured in the subjects (typical waveforms were shown in Figure 1; analysis procedure was shown in Figure 2). Frequency-domain analysis was applied to derive the 40 harmonic indices from the measured BPW signal (*n* = 1–10): amplitude proportion (*C_n_*), coefficient of variation of *C_n_* (*CV_n_*), phase angle (*P_n_*), and standard deviation of *P_n_* (*P_n_*_*SD*) (details of measurement and analysis are listed in Supplemental Materials). The present study used the MMSE and machine-learning analysis (eight models; models details see Table 1) to investigate whether measured pulse indices are related to the cognitive condition in a sample of 38 AD patients, 38 control subjects, 39 community subjects, and 8 young subjects (see Table 2). The eight machine-learning methods used in the present study included support vector machine (SVM), multilayer perception (MLP), Gaussian Naive Bayes (GNB), decision tree (DT), random forest (RF), logistic regression (LR), linear discriminant analysis (LDA), and K-nearest neighbor classification (KNN). When performing the threefold cross validation of the training stage, we first randomly assigned the subjects into three subgroups, and then the pulse sequence of the subjects within each subgroup were used to train the model. When performing the testing stage, the data sequence of the pulse indices of the subject was input into the trained model to get the classification probability.

The subjects were recruited from the Ren-Ai Branch of Taipei City Hospital. Informed consent was obtained from the study participants or their legal designates (approved by the Review Board of Taipei City Hospital; approval no. TCHIRB-10810016-E). A neurologist or psychiatrist diagnosed AD, and evaluated the severity of disability in patients with dementia [11]. Community subjects were recruited at two sites: Site A was Taipei Veterans Home, located in the countryside of New Taipei City, and Site B was Hoping LOHAS Daycare Center, located near the educational area of Taipei (near to National Taiwan University and National Taiwan Normal University). Eight graduate students of National Taiwan University of Science and Technology were also recruited as the young group. The study was approved by the Research Ethics Committee, National Taiwan University (approval no. 202010EM001). Based on MMSE scores, the subjects were categorized into mild dementia (MMSE scores > 16 and ≤24), moderate dementia (MMSE scores > 10 and ≤16), and severe dementia (MMSE scores ≤ 10). Subjects were excluded if they did not agree to participate in the study or were unable to cooperate with the research steps, such as due to their limbs trembling involuntarily, restlessness, or agitated movements.

## 3. Results

**Figure 3:**
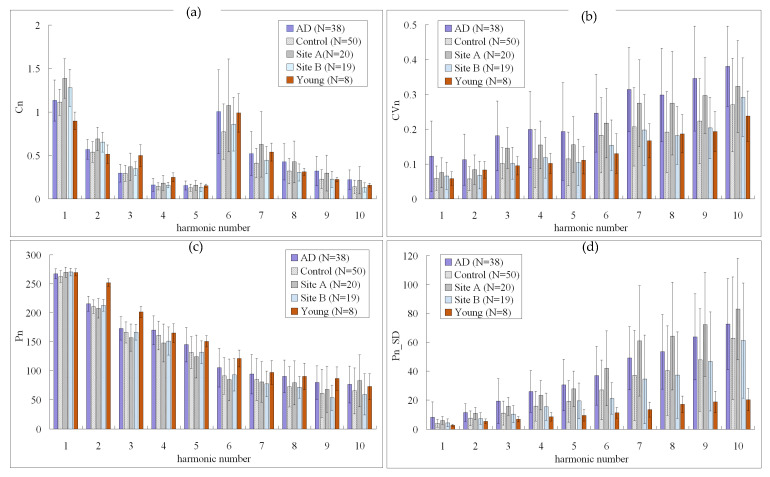
Comparisons of BPW harmonic indices of AD patients, control, community (Sites A and B), and young subjects: (**a**) *C_n_*, (**b**) *CV_n_*, (**c**) *P_n_*, and (**d**) *P_n_*_*SD*. Data are mean and standard-deviation values. *C*_6_–*C*_10_ values have been multiplied by 10 to make the differences clearer. *p* values are listed in Table 3.

The characteristics of the study subjects are listed in Table 2. Figure 3 compares the harmonic indices of the BPW signals (*p* values are listed in Table 3). For the amplitude ratios, *C*_4_–*C*_10_ were larger in the AD patients than the control subjects (significantly for *C*_5_–*C*_10_). All *C_n_* indices were larger in Site-A subjects than in Site-B subjects (significantly for *C*_8_ and *C*_10_). For phase-angle indices of BPW signals, Group AD had the largest values of all *CV_n_* indices and *P*_2_–*P*_9_ (compared with Control; *p* < 0.05 for *P*_8_ and *P*_9_, 0.05 < *p* < 0.1 for *P*_5_ and *P*_6_).

For variability indices of BPW signals, Group AD had the largest values of all *CV_n_* indices and *P_n__SD*_1_ to *P_n__SD*_5_. Group AD had larger values than Group Control of all *CV_n_* indices (all significant) and *P_n__SD* indices (all significant except for *P_n__SD*_10_). Site A had larger values than Site B of all *CV_n_* indices (*p* < 0.05 for *CV*_3_, *CV*_5_, *CV*_6_, *CV*_8_, and *CV*_9_; 0.05 < *p* < 0.1 for *CV*_4_ and *CV*_7_) and *P_n__SD* indices (*p* < 0.05 for *P_n__SD*_2_ to *P_n__SD*_10_, 0.05 < *p* < 0.1 for *P_n__SD*_1_). Group Young had smaller values than Group AD of all *P_n__SD* indices among the groups (all significant except for *P_n__SD*_1_).

**Figure 4:**
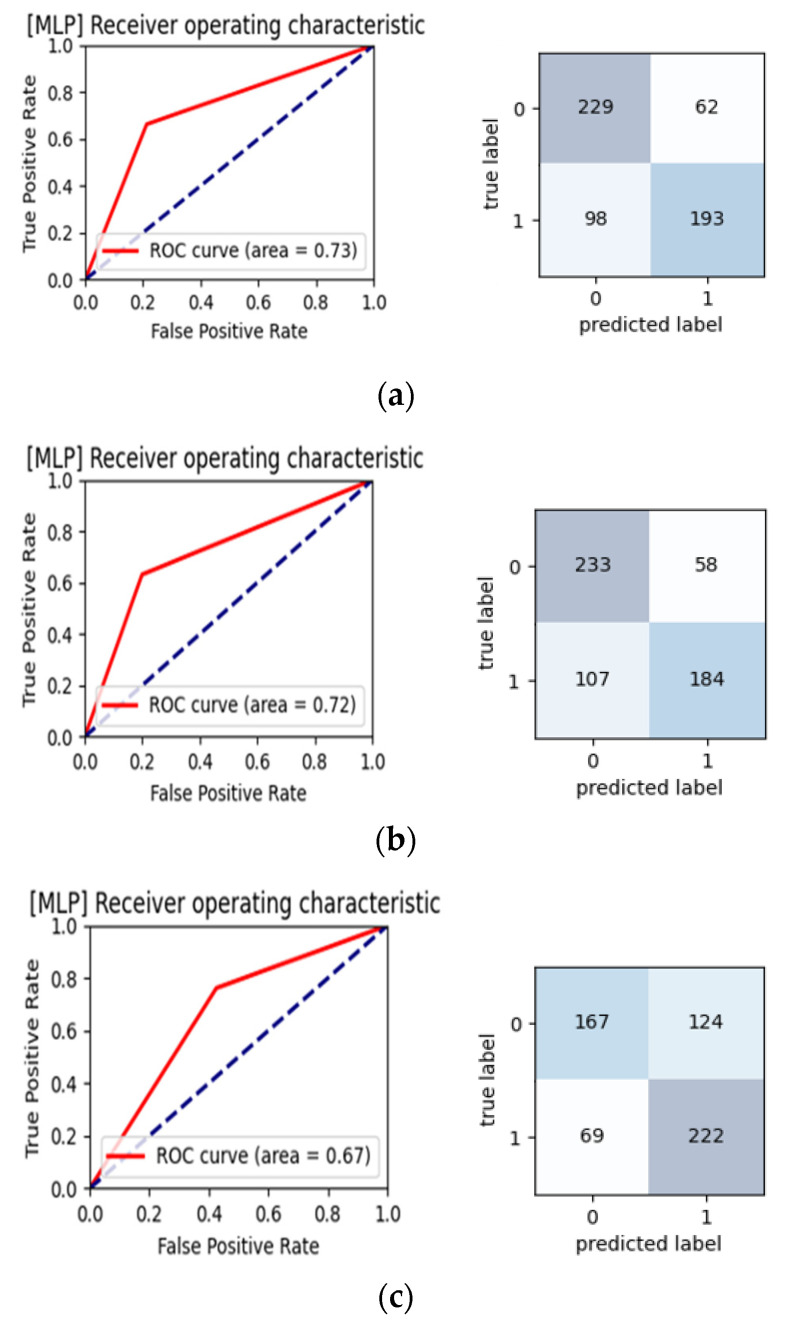
MLP analysis results for comparisons of BPW indices between AD patients and Group Control. Training and validation accuracy plots, AUC, and contradiction matrix are presented for the threefold cross-validation. The mean accuracy, sensitivity, specificity, and AUC were 70.32%, 0.68, 0.72, and 0.70, respectively. “1” indicates AD patients and “0” indicates Control. (**a**) 1st part; (**b**) 2nd part; (**c**) 3rd part of the threefold cross-validation.

Table 4 lists the machine-learning analysis results (accuracy, sensitivity, specificity, and AUC) for evaluating the performance in classifying the subjects into the AD and Control groups. MLP had the best AUC (70.32%) among the eight methods. Detailed results of the threefold cross-validation analysis for MLP are shown in Figure 4.

**Figure 5:**
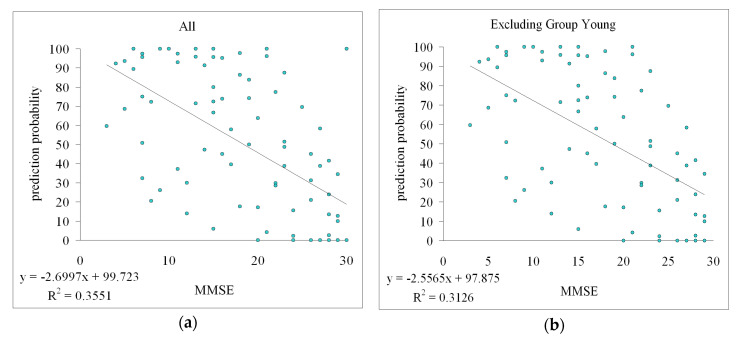
Correlation between the prediction probability and MMSE score. Group AD and Control were used as training data. Community subjects at Sites A and B, and Group Young were used as test subjects. (**a**), There was a significant negative correlation for the testing community subjects (*R*^2^ = 0.36, *p* < 0.05 by *F*-test). (**b**), When the young group was excluded, there was still a significant negative correlation (*R*^2^ = 0.31, *p* < 0.05 by *F*-test).

The correlations found in the testing results between the prediction probability (using AD patients and Control as training data) and the MMSE scores for the community and young subjects are shown in Figure 5. There was a significant negative correlation for these testing subjects (*R*^2^ = 0.36, *p* < 0.05 by *F*-test). When the young group was excluded to minimize the possible interference effects of different ages, there was still a significant negative correlation for the community subjects (*R*^2^ = 0.31, *p* < 0.05 by *F*-test).

## 4. Discussion

The present study found significant differences in BPW spectral indices between AD patients and control subjects. Using AD patients and control subjects as training data, a significant correlation was found between the prediction probability of the test data (comparing community subjects at two sites and young subjects) and the MMSE score.

### 4.1. Differences in the Spectral Indices of the Pulse Waveform

Differences in the BPW spectral indices between AD patients and control subjects were similar to those noted in our previous study [11]. Figure 3 reveals that *C*_5_–*C*_10_ were significantly larger for AD patients than controls. Similarly for the subjects at the two community sites, *C_n_* values were larger in Site-A subjects than in Site-B subjects (significant for *C*_8_ and *C*_10_). Site B is located in the educational area of the city, whereas Site A is located in the countryside, and so Site-B subjects are probably more likely to experience diverse kinds of cognitive stimulation, therefore leading differences in cognitive function between the subjects at the two sites. This conjecture is supported by the difference in the MMSE scores between the two sites: although not statistically significant, the MMSE score was slightly lower in Site-A subjects (21.84 ± 5.19) than in Site-B subjects (23.95 ± 4.39).

It has been demonstrated previously that dementia can occur in association with an increase in the arterial stiffness [4,5]. This implies that it is possible for dementia to be accompanied with changes in the arterial pulse transmission condition outside the cerebrovascular vascular system, and hence measuring and analyzing the pulse waveform acquired at some distal site could be used to aid the evaluation of dementia-induced vascular changes in the pulse waveform. It has also been suggested that the larger *C_n_* values of dementia patients can be partly attributed to the increased transmission efficiency for the higher-frequency components of the pulse spectrum [11]. The present findings of cognitive function differing between subjects at different community sites suggest that this is associated with changes in the vascular stiffness that affect the arterial pulse wave transmission and hence change the *C_n_* values. The MMSE scores of AD patients (12.16 ± 5.52) were closer to those of Site-A subjects than Site-B subjects, which may therefore be associated with larger *C_n_* values for several higher-frequency components of the pulse spectrum.

Regarding variability indices, Figure 3 indicates that AD patients had the largest values of all *CV_n_* indices (all significant compared with Control) and many *P_n__SD_n_* indices (significant for *P_n__SD*_1_ to *P_n__SD*_9_ compared with Control). Variability indices such as HR and BP variability have been used in many studies to aid the monitoring of cardiovascular regulatory activities [21]. Variability indices of the pulse waveform in AD patients have previously been suggested to illustrate the presence of larger regulatory activities acting on vascular elastic properties [11]. This could be related to the greater effort needed to address the changes in the blood-flow perfusion condition when facing AD-induced changes in vascular stiffness.

Similar to the situation for *C_n_* indices, since the values of many of the analyzed pulse variability indices were significantly larger for AD patients, comparison between Site-A and Site-B subjects revealed that those at Site A had larger values of all *CV_n_* indices (some of them were significant) and *P_n__SD* indices (significant for *P_n__SD*_2_ to *P_n__SD*_10_). Based on the above-mentioned conjecture, the differences in the cognitive function of subjects between the two sites may induce different vascular regulatory activities, and hence may partly account for the observed differences in the *CV_n_* and *P_n__SD_n_* indices.

Another finding supporting this conjecture is that Group Young had the smallest values of all *P_n__SD* indices (significant for *P_n__SD*_2_ to *P_n__SD*_10_ compared with AD patients). The MMSE scores of these young subjects were higher than those of Site-A and Site-B subjects. Based on the above-mentioned conjecture, the regulatory efforts may be smallest due to the high cognitive function of the young subjects. The vascular regulatory activities of Group Young may therefore be smallest, hence leading to the smallest values of *P_n__SD* indices.

Regarding phase-angle indices of BPW signals, Group AD had the largest values of *P*_2_–*P*_9_ (significant for *P*_8_ and *P*_9_ compared with Control). The phase angle is related to the starting time point for each frequency component. A larger *P_n_* value can therefore be partly attributed to faster propagation of that specific frequency component of the arterial pulse, and hence related to the spectral distribution of the vascular elasticity (increased vascular elasticity for some specific frequency components).

### 4.2. Correlation between Prediction Probability and MMSE Score

Further important support for the above-mentioned conjecture comes from the correlation between the MMSE score and the prediction probability identified in the machine-learning analysis using spectral pulse indices as features. While there have been advances in detecting early neuropathology, it may be necessary to consider a shift in the diagnostic paradigm so that milder dementia can be detected earlier in order to obtain greater benefits from interventions [22,23]. Identifying the symptoms of the early stages of dementia is often difficult among older adults living in residential care [24]. It has been reported that more than 10% of community-dwelling subjects older than 70 years suffer from very mild or mild dementia [22]. Data-informed decision-making strategies to identify individuals at high risk of dementia could be essential to facilitating large-scale prevention and early intervention [25]. Triage tests such as the MMSE are used in clinical practice to rapidly assess the cognitive condition [2]. We therefore used the MMSE in comparisons with the results of machine-learning pulse analysis in the present study.

In previous community studies, the diagnostic accuracy of MMSE was indicated by a sensitivity of 0.85 (95% CI = 0.74–0.92) and a specificity of around 0.90 (95% CI = 0.82–0.95) [1]. A previous study that applied machine-learning algorithms used the MMSE, the Montreal Cognitive Assessment, and the Korean Dementia Screening Questionnaire to evaluate participants, and achieved an overall screening accuracy of >90% for mild cognitive impairment, dementia, and cognitive dysfunction [26]. Although the MMSE has the largest body of evidence to support its use and has adequate test accuracy, its utility is limited by the relatively long administration time (10–15 min) and high cost [2]. The present results (Figure 4) indicated an AUC of 0.70 in the threefold cross-validation when using MLP, which represents acceptable discrimination performance.

The significant correlation between the prediction probability and the MMSE score noted in the present study provides further support for possible application in community subjects. Figure 5 indicates that a higher MMSE score was associated with a lower prediction probability. This illustrates that there could be a physiological connection between the MMSE evaluation and the pulse indices; that is, when the MMSE score is lower (which indicates worse cognition), the prediction probability is higher (indicating greater similarity of the pulse waveforms between the subject and the average of the AD group), and vice versa.

The young group was included in the test subjects for the data shown in Figure 5a. Subjects of different ages may exhibit different levels of vascular stiffness [27], which could interfere with arterial pulse transmission and hence the pulse indices. To elucidate the relationship between the prediction probability and the MMSE score, the young group was excluded in Figure 5b to minimize the possible effects of different ages in the comparisons. After removing these data points of the young group, there was still a significant correlation (with *R*^2^ changing from 0.36 to 0.31). This illustrated that even when the age varied between groups of testing data, the correlation between the prediction probability and the MMSE score remained statistically significant.

Since the MMSE is a widely used tool for evaluating cognitive function in community subjects, the present finding of a significant correlation illustrates a possible connection of underlying physiological mechanisms between arterial pulse transmission and the MMSE score. Other efforts have been made to identify possible connections between physiological measurements and cognition evaluation indices. For example, one previous study focused on the activities of daily living of adults in a smart-home setting to monitor potential cognitive anomalies using a public data set, and achieved a 90.74% accuracy in detecting the onset of dementia by applying machine-learning analysis [24]. In the present study, the pulse data took only 3 min to acquire; this shorter administration time enhances the user-friendliness of the present method of pulse-wave measurements for discriminating cognitive conditions, and hence represents a potential method for the early screening of dementia.

This study was limited by the relatively small sample in the machine-learning analysis (although cross-validation was used). Future efforts could focus on accumulating more patients and community subjects in order to verify the present conjectures.

## 5. Conclusions

The findings of this study and the related conclusions to be drawn can be summarized as follows:

∎ Significant differences in spectral indices of the BPW were found between the AD patients and control subjects.
∎ The threefold cross-validation results indicated an AUC of 0.70 in the threefold cross-validation when using MLP, which indicated acceptable discrimination performance.
∎ Using AD patients and control subjects as training data, a significant correlation was found between the prediction probability of the test data (comprising community subjects at two sites and young subjects) and the MMSE score. Although significant, the correlation in Figure 5 was modestly correlated. Further collection of subject data in future work is necessary to strengthen the present conjecture.
∎ Age did not markedly interfere with the identified correlation between the prediction probability and the MMSE score.
∎ The present findings based on pulse waveform measurements and machine-learning analysis may be meaningful for the development of a noninvasive, rapid, and objective method for monitoring the cognitive condition.

## Figures and Tables

**Table 1:** Parameters of the machine-learning models.

Machine-Learning Methods	Model Parameters
SVM (support vector machine)	C = 1; kernel: rbf; gamma: auto; tol = 0.0001; max_iter = −1; class_weight: none
MLP (multilayer perception)	hidden_layer_sizes = 100; solver: adam; alpha = 0.0001; batch_size: auto; max_iter = 200; learning_rate_int = 0.001
GNB (Gaussian Naive Bayes)	Priors: none
DT (decision tree)	Criterion: gini; Splitter: best; max_depth: none; min_samples_split = 2; min_samples_leaf = 1; min_weight_fraction_leaf = 0; max_features: none; max_leaf_nodes: none; min_impurity_split = 0.0
RF (random forest)	n_estimators = 100; criterion: gini; max_depth: none; min_samples_split = 2; min_samples_leaf = 1; min_weight_fraction_leaf = 0; max_features: none; max_leaf_nodes: none
LR (logistic regression)	Penalty: l2; Solver: lbfgs; multi_class: auto; class_weight: none
LDA (linear discriminant analysis)	Solver: svd; Shrinkage: none; Priors: none
KNN (K-nearest neighbor classification)	n_neighbors = 5; weights: uniform; algorithm: auto; n_jobs: none; p: none

**Table 2:** Characteristics of subjects.

	**AD patients**					
	Mild dementia 16 < MMSE < 24		Moderate dementia 10 < MMSE ≤ 16		Heavy dementia MMSE ≤ 10	
gender	male	female	male	female	male	female
Subject number	4	6	5	7	6	10
subject number (male + female)	10		12		16	
Total subject number	38					
Age	71.33 ± 6.5	73.86 ± 7.86	67 ± 19	77.42 ± 11.51	74.33 ± 9.29	77.4 ± 7.02
Age(male + female)	73.1 ± 7.21		73.08 ± 15.27		76.25 ± 7.79	
Age (all)	74.42 ± 10.44					
HR	68 ± 11.53	70.14 ± 11.86	67 ± 3.53	67.85 ± 16.24	66.4 ± 13.92	67.6 ± 9.64
HR (male + female)	69.5 ± 11.57		67.5 ± 12.19		68.87 ± 11.1	
HR (all)	68.8 ± 11.18					
	**Community Site A (Taipei Veterans Home)**					
	MMSE > 24		Mild dementia 16 < MMSE < 24		Moderate dementia 10 < MMSE ≤ 16	
gender	male	female	male	female	male	female
Subject number	8	0	7	0	5	0
subject number (male + female)	8		7		5	
Total subject number	20					
Age	81.09 ± 10.31		83.43± 9.02		77.08 ± 5.36	0
Age(male + female)	81 ± 10.31		83± 9.02		86.4 ± 7.92	
Age (all)	83.05 ± 9.10					
HR	67.25 ± 15.26		68.29 ± 4.72		62.20 ± 5.22	
HR (male + female)	67.25 ± 15.26		68.29 ± 4.72		62.20 ± 5.22	
HR (all)	66.35 ± 10.43					
	**Community Site B (Hoping LOHAS Daycare Center)**					
	MMSE > 24		Mild dementia 16 < MMSE ≤ 24		Moderate dementia 10 < MMSE ≤ 16	
gender	male	female	male	female	male	female
Subject number	2	8	1	6	2	0
subject number (male + female)	10		7		2	
Total subject number	19					
Age	71.53 ± 0.71	75.64± 6.97	76.23	81.26 ± 4.51	84.46 ± 6.36	
Age(male + female)	74.3 ± 6.33		80.71± 4.61		84.46 ± 6.36	
Age (all)	78.25 ± 6.88					
HR	79.50 ± 12.02	68.38 ± 6.86	61.00	67.00 ± 8.00	65.50 ± 6.36	
HR (male + female)	70.6 ± 8.64		66.14 ± 7.65		65.50 ± 6.36	
HR (all)	68.42 ± 8.04					
	**Control**		**Young**			
gender	male	female	male	female		
Subject number	11	27	7	1		
Total subject number	38		8			
Age	74.24 ± 3.26	72.08 ± 4.94	23.85 ± 1.46	23		
Age (all)	72.71 ± 4.58		23.75 ± 1.38			
HR	78.09 ± 9.11	79.88 ± 7.27	66.00 ± 5.94	64.00		
HR (all)	79.36 ± 7.76		65.75 ± 5.54			

**Table 3:** Probability values for comparisons of BPW harmonic indices (*C_n_*, *CV_n_*, *P_n_*, and *P_n_*_*SD*) between AD patients, controls, and community subjects. Significant differences were underlined.

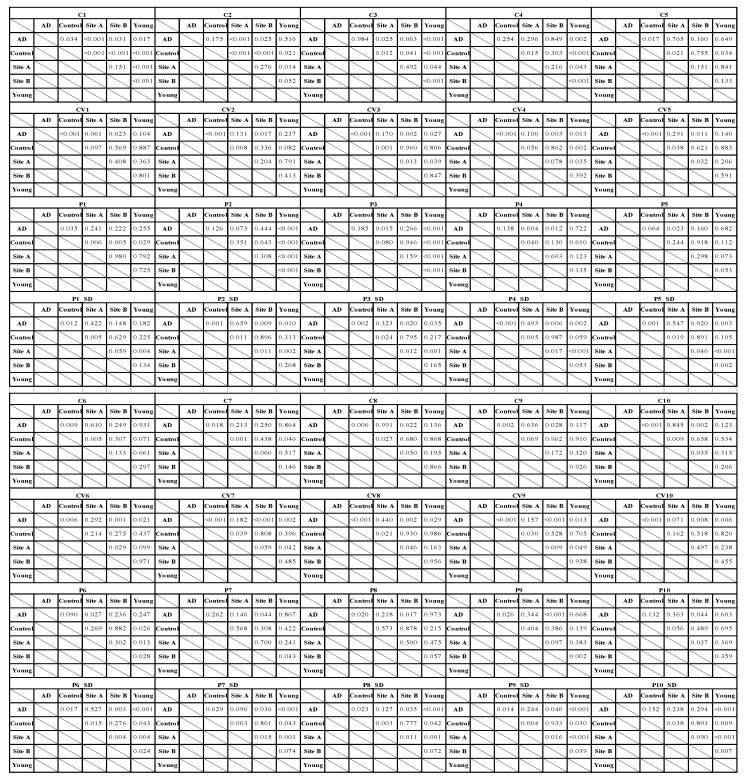

**Table 4:** Results of the machine-learning analyses comparing BPW indices between AD patients and Control. Results are presented for the threefold cross-validation.

Accuracy (%)	SVM	MLP	GNB	DT	RF	LR	LDA	KNN
1	70.61	72.50	61.34	63.57	64.26	71.47	76.80	64.94
2	56.35	71.64	55.84	64.77	69.41	62.37	71.64	62.37
3	60.30	66.83	60.48	59.79	63.40	62.71	56.87	63.91
average	62.42	70.32	59.22	62.71	65.69	65.52	68.44	63.74
Sensitivity	SVM	MLP	GNB	DT	RF	LR	LDA	KNN
1	0.66	0.66	0.38	0.61	0.72	0.61	0.64	0.60
2	0.46	0.63	0.21	0.62	0.71	0.47	0.61	0.51
3	0.78	0.76	0.75	0.81	0.91	0.77	0.68	0.78
average	0.63	0.68	0.45	0.68	0.78	0.62	0.64	0.63
Specificity	SVM	MLP	GNB	DT	RF	LR	LDA	KNN
1	0.74	0.78	0.84	0.65	0.56	0.81	0.89	0.69
2	0.66	0.80	0.90	0.66	0.67	0.77	0.81	0.73
3	0.41	0.57	0.45	0.37	0.35	0.48	0.45	0.49
average	0.60	0.72	0.73	0.56	0.53	0.69	0.72	0.64
AUC	SVM	MLP	GNB	DT	RF	LR	LDA	KNN
1	0.70	0.72	0.61	0.63	0.64	0.71	0.76	0.64
2	0.56	0.71	0.55	0.64	0.69	0.62	0.71	0.62
3	0.60	0.66	0.60	0.59	0.63	0.62	0.56	0.63
average	0.62	0.70	0.59	0.62	0.65	0.65	0.68	0.63

## Data Availability

The data presented in this study are available on request from the corresponding author. The data are not publicly available due to ethical concern.

## Supplementary Materials

The following supporting information can be downloaded at: https://www.mdpi.com/article/10.3390/s22030806/s1.

## References

[1] Creavin S.T., Wisniewski S., Noel-Storr A.H., Trevelyan C.M., Hampton T., Rayment D., Thom V.M., Nash K.J., Elhamoui H., Milligan R. (2016). Mini-Mental State Examination (MMSE) for the detection of dementia in clinically unevaluated people aged 65 and over in community and primary care populations. Cochrane Database Syst. Rev..

[2] Patnode C.D., Perdue L.A., Rossom R.C., Rushkin M.C., Redmond N., Thomas R.G., Lin J.S. (2020). Screening for Cognitive Impairment in Older Adults: Updated Evidence Report and Systematic Review for the US Preventive Services Task Force. JAMA.

[3] Dequanter S., Buyl R., Fobelets M. (2020). Quality indicators for community dementia care: A systematic review. Eur. J. Public Health.

[4] Iadecola C., Gottesman R.F. (2018). Cerebrovascular alterations in Alzheimer disease: Incidental or pathogenic?. Circ. Res..

[5] Cortes-Canteli M., Iadecola C. (2020). Alzheimer’s disease and vascular aging: JACC Focus Seminar. J. Am. Coll. Cardiol..

[6] Kuller L.H., Lopez O.L., Mackey R.H., Rosano C., Edmundowicz D., Becker J.T., Newman A.B. (2016). Subclinical cardiovascular disease and death, dementia, and coronary heart disease in patients 80+ years. J. Am. Coll. Cardiol..

[7] O’Rourke M.F., Adji A., Safar M.E. (2018). Structure and Function of Systemic Arteries: Reflections on the Arterial Pulse. Am. J. Hypertens..

[8] Wilkinson I.B., Cockcroft J.R., Webb D.J. (1998). Pulse wave analysis and arterial stiffness. J. Cardiovasc. Pharmacol..

[9] Oh Y.S. (2018). Arterial stiffness and hypertension. Clin. Hypertens..

[10] Hsiu H., Liu J.C., Yang C.J., Chen H.S., Wu M.S., Hao W.R., Lee K.Y., Hu C.J., Wang Y.H., Fang Y.A. (2021). Discrimination of vascular aging using the arterial pulse spectrum and machine-learning analysis. Microvasc. Res..

[11] Lin S.K., Hsiu H., Chen H.S., Yang C.J. (2021). Classification of patients with Alzheimer’s disease using the arterial pulse spectrum and a multilayer-perceptron analysis. Sci. Rep..

[12] Husmann M., Jacomella V., Thalhammer C., Amann-Vesti B.R. (2015). Markers of arterial stiffness in peripheral arterial disease. Vasa.

[13] Mackenzie I.S., Wilkinson I.B., Cockcroft J.R. (2002). Assessment of arterial stiffness in clinical practice. QJM.

[14] Liao J., Farmer J. (2014). Arterial stiffness as a risk factor for coronary artery disease. Curr. Atheroscler Rep..

[15] Lin F.C., Hsiu H., Chiu H.S., Chen C.T., Hsu C.H. (2020). Characteristics of pulse-waveform and laser-Doppler indices in frozen-shoulder patients. Biomed. Signal Process. Control.

[16] Chen C.T., Hsiu H., Hung S.H., Chen G.Z., Huang Y.L. (2017). Characteristics of spectral indexes of the blood pressure waveform in patients with breast cancer. Blood Press. Monit..

[17] Chang Y.W., Hsiu H., Yang S.H., Fang W.H., Tsai H.C. (2016). Characteristics of beat-to-beat photoplethysmography waveform indexes in subjects with metabolic syndrome. Microvasc. Res..

[18] Hsu C.L., Hsiu H., Hsu W.C., Chen C.Y. (2014). Characteristics of harmonic indexes of the arterial blood pressure waveform in polycystic ovary syndrome. Blood Press Monit..

[19] Chen C.T., Ting C.T., Chen C.Y., Lyu Z.J., Chen C.C., Chou Y.S., Cheng C.F., Hsu C.H., Hsiu H. (2020). Pulse-waveform and laser-Doppler indices for identifying colorectal-cancer patients. Biom. Eng. Appl. Basis Comm..

[20] Sorelli M., Perrella A., Bocchi L. (2018). Detecting vascular age using the analysis of peripheral pulse. IEEE Trans. Biomed. Eng..

[21] Stergiou G.S., Ntineri A., Kollias A., Ohkubo T., Imai Y., Parati G. (2014). Blood pressure variability assessed by home measurements: A systematic review. Hypertens. Res..

[22] Lam L.C., Tam C.W., Lui V.W., Chan W.C., Chan S.S., Wong S., Wong A., Tham M.K., Ho K.S., Chan W.M. (2008). Prevalence of very mild and mild dementia in community-dwelling older Chinese people in Hong Kong. Int. Psychogeriatr..

[23] Trivedi D. (2017). Cochrane Review Summary: Mini-Mental State Examination (MMSE) for the detection of dementia in clinically unevaluated people aged 65 and over in community and primary care populations. Prim. Health Care Res. Dev..

[24] Ahamed F., Shahrestani S., Cheung H. (2020). Internet of Things and Machine Learning for Healthy Ageing: Identifying the Early Signs of Dementia. Sensors.

[25] Luo H., Lau K.K., Wong G.H.Y., Chan W.C., Mak H.K.F., Zhang Q., Knapp M., Wong I.C.K. (2020). Predicting dementia diagnosis from cognitive footprints in electronic health records: A case-control study protocol. BMJ Open.

[26] Yim D., Yeo T.Y., Park M.H. (2020). Mild cognitive impairment, dementia, and cognitive dysfunction screening using machine learning. J. Int. Med. Res..

[27] Faconti L., Bruno R.M., Ghiadoni L., Taddei S., Virdis A. (2015). Ventricular and vascular stiffening in aging and hypertension. Curr. Hypertens. Rev..

