# Supplementary material for: Discrimination of the Cognitive Function of Community Subjects Using the Arterial Pulse Spectrum and Machine-Learning Analysis
Source: Sensors (Basel). 2022 Jan 21;22(3):806. doi: 10.3390/s22030806 (PMC8838619; doi:10.3390/s22030806)
Supplement: Supplementary file 1 [file sensors-22-00806-s001.zip › sensors-1518268-supplementary.pdf]

1. Measurements

Before the measurements, the subjects were relaxed and rested for 20 minutes. For each experiment, the subjects were sitting on a chair, and we recorded a 3-minute data sequence. The BPW signal was acquired by a pressure transducer (KFG-2-120-D1-11, Kyowa) held onto the skin surface above the radial artery 2 cm from the left wrist, and was sampled at 1024 Hz [11, 12, 16]. Before the measurement, the heart rate (HR), brachial systolic BP and diastolic BP were measured by using a sphygmomanometer (MG150f, Rossmax).

2. Analysis

The present analysis procedure included signal processing and information processing:

- signal processing

Frequency-domain analysis was applied to derive the 40 harmonic indices from the measured BPW signal (n=1-10): amplitude proportion (C_n), coefficient of variation of C_n (CV_n), phase angle (P_n), and standard deviation of P_n (P_n_SD).

Each individual pulse (between foot points) can be represented by the following finite series [11, 12, 16]. The pulses were excluded if the values between the two foot points were larger than 20% of the pulse amplitude.

$$x(t) = \frac{A_0}{2} + \left\{ \sum_{n=1}^{k/2} A_n \cos n\omega t_s + \sum_{n=1}^{k/2} B_n \cos n\omega t_s \right\}$$

The Fourier coefficients (A_n and B_n) of the pulse can be calculated as

$$A_n = \frac{2}{k} \sum_{s=0}^{k} x_s \cos n\omega t_s \quad (for\ n = 0,1,...,\frac{k}{2})$$

$$B_n = \frac{2}{k} \sum_{s=0}^{k} x_s \sin n\omega t_s \quad (for\ n = 0,1,...,\frac{k}{2})$$

where ω is the angular frequency and t_s is the sampling time interval.

The amplitude (Amp_n) and phase angle (P_n) of each harmonic of the pulse harmonic spectrum can then be calculated as $Amp_n = \sqrt{A_n^2 + B_n^2}$ and $P_n = \arctan(B_n / A_n)$. The amplitude proportions (C_n values) for each pulse were calculated as $Amp_n / Amp_0 \times 100\%$, for

n = 1–10. CV_n was then calculated as the coefficient of variations of C_n, and P_n_SD was calculated as the standard deviation of P_n.

Signal processing was performed with MATLAB (MathWorks). The differences were considered significant when $p<0.05$; all p-values were two-sided hypotheses. Kruskal–Wallis test (nonparametric ANOVA) was used to compare the indices between groups. Post hoc multiple comparisons tests were made by Dunn's test.

- information processing (procedure shown in Fig.2)

For information processing, the features of pulse signals were collected from the results of the signal-processing stage described above, to yield 40 indices for each pulse: C_n, CV_n, P_n, and P_n_SD values for n = 1–10. Each feature was scaled by Z-score normalization to eliminate the effects of the variations in the ranges of different indices [11, 12]. Python (version 3.7) was used as the analysis tool in the information processing; eight machine-learning methods were used to classify the data (details of model parameters are listed in Table 2) [12].

For the AD patients and control subjects, threefold cross validation was used in the model training stage. The proposed classification model was evaluated by calculating the accuracy, AUC (area under the receiver operating characteristics curve), sensitivity and specificity.

In the testing stage, the 40 features of each community subject (Sites A and B, and young Group) were input into the trained algorithm to get the prediction probability; 50% probability was used as the classification criteria. The prediction probability was plotted versus MMSE score and the linear regression was performed to study the possible relation between them.
